# Supplementary material for: Simultaneous Expression of Th1- and Treg-Associated Chemokine Genes and CD4+, CD8+, and Foxp3+ Cells in the Premalignant Lesions of 4NQO-Induced Mouse Tongue Tumorigenesis
Source: Cancers (Basel). 2021 Apr 12;13(8):1835. doi: 10.3390/cancers13081835 (PMC8069711; doi:10.3390/cancers13081835)
Supplement: Supplementary file 1 [file cancers-13-01835-s001.pdf]

cancers

MDPI

Supplementary

Simultaneous Expression of Th1- and Treg-Associated Chemokine Genes and Infiltration of CD4$^+$, CD8$^+$, and Foxp3$^+$ Cells in the Premalignant Lesions of 4NQO-induced Mouse Tongue Tumorigenesis

Hana Yamaguchi et al.

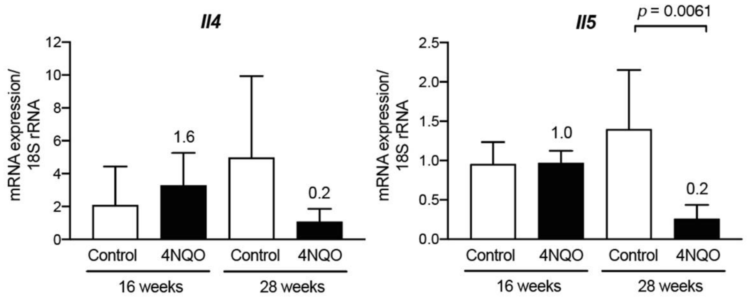

FigureS1. *Il4* and *Il5* transcript levels in tongue tissues from 4NQO-treated mice. mRNA levels of *Il4* and *Il5* in the tongue tissues of mice treated with vehicle (control) or 4NQO were assessed at the indicated time points by qRT-PCR. Relative mRNA levels were normalized to 18S rRNA. Each column and bar represents the mean ± SD (n = 6). Statistically significant differences in mRNA expression in 4NQO-treated mice relative to control mice are indicated. Fold induction of mRNA expression in tongue tissues from mice treated with 4NQO relative to that in control mice is shown above the column.

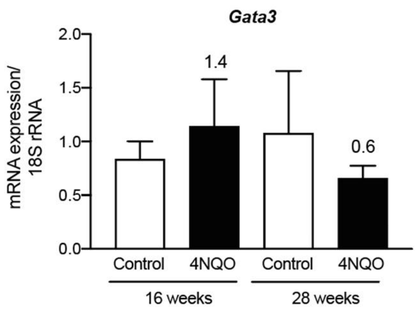

Figure S2. *Gata3* transcript levels in tongue tissues from 4NQO-treated mice. The mRNA levels of *Gata3* in the tongue tissues of mice treated with vehicle (control) or 4NQO were assessed at the indicated time points by qRT-PCR. Relative mRNA levels were normalized to 18S rRNA. Each column and bar represents the mean ± SD (n = 6). Fold induction of mRNA expression in the tongue tissue from mice treated with 4NQO compared to that in control mice is shown above the column.

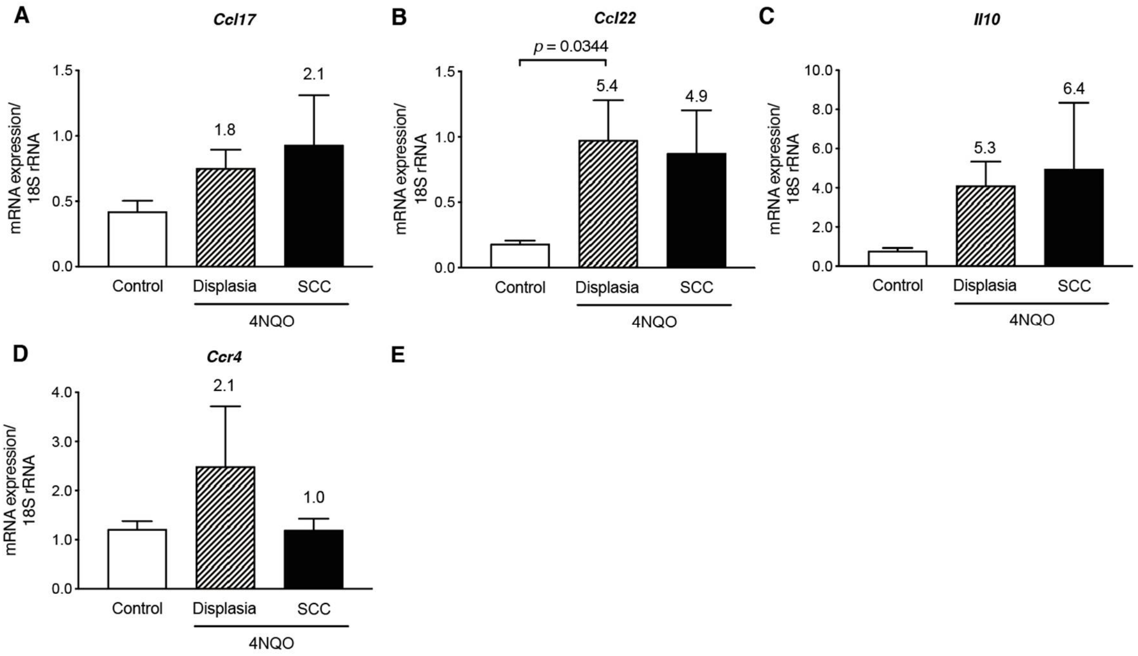

Figure S3. The transcript levels of Th2/Treg-associated chemokines/cytokines and *Ccr4* in dysplasia and SCC tongue tissue at 28 weeks after 4NQO treatment. mRNA levels in the tongue tissue of control mice or in the dysplasia or SCC lesions were assessed by qRT-PCR: *Ccl17* (**A**); *Ccl22* (**B**); *Il10* (**C**); and *Ccr4* (**D**). Relative mRNA levels were normalized to 18S rRNA. Each column and bar represents the mean and SD (n = 6) for the control and for dysplasia and SCC mice (n = 3 each). Statistically significant differences in the mRNA expression of 4NQO-treated mice compared to control mice are indicated. Fold induction of mRNA expression in the tongue tissues from mice treated with 4NQO compared to that in control mice is shown above the column.

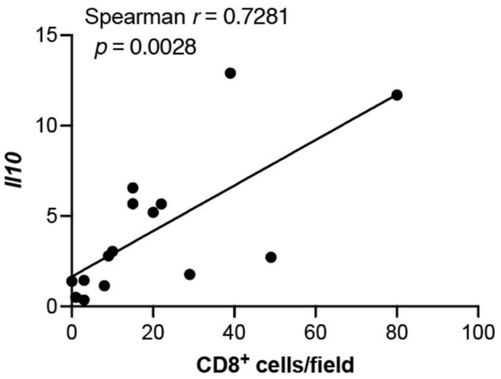

Figure S4. Correlations between transcript levels of *Il10* and CD8^{+} cells in tongue tissues from 4NQO-treated mice. mRNA levels of *Il10* in tongue tissues from each mouse were assessed for correlations with infiltrated CD8^{+} cells via Spearman's rank correlation coefficient analysis.

Table S1. Gene lists for the RT^2 Profiler PCR Array.

Position	Unigene	Refseq	Symbol	Description	Gname	RT2 Catalog
A01	Mm.3969	NM_009605	Adipoq	Adiponectin, C1Q and collagen domain con	30kDa/APN/Acdc/Acrp30/GBP28/adipo/apM1	PPM05260A
A02	Mm.103205	NM_007553	Bmp2	Bone morphogenetic protein 2	AI467020/Bmp2a	PPM03753A
A03	Mm.6813	NM_007554	Bmp4	Bone morphogenetic protein 4	Bmp-4/Bmp2b/Bmp2b-1/Bmp2b1	PPM02998F
A04	Mm.385759	NM_007556	Bmp6	Bone morphogenetic protein 6	D13Wsu115e/Vgr1	PPM04429F
A05	Mm.392150	NM_007557	Bmp7	Bone morphogenetic protein 7	OP1	PPM03001C
A06	Mm.1283	NM_011329	Ccl1	Chemokine (C-C motif) ligand 1	BF534335/I-309/Scya1/Tca-3	PPM03138C
A07	Mm.4686	NM_011330	Ccl11	Chemokine (C-C motif) ligand 11	Scya11/eotaxin	PPM02967G
A08	Mm.867	NM_011331	Ccl12	Chemokine (C-C motif) ligand 12	MCP-5/Scya12	PPM02977E
A09	Mm.41988	NM_011332	Ccl17	Chemokine (C-C motif) ligand 17	Abcd-2/Scya17/Scya17l/Tarc	PPM02963B
A10	Mm.490604	NM_011888	Ccl19	Chemokine (C-C motif) ligand 19	CKb11/ELC/MIP3B/Scya19/exodus-3	PPM03157C
A11	Mm.290320	NM_011333	Ccl2	Chemokine (C-C motif) ligand 2	AI323594/HC11/JE/MCAF/MCP-1/MCP1/SMC-CF	PPM03151G
A12	Mm.116739	NM_016960	Ccl20	Chemokine (C-C motif) ligand 20	CKb4/LARC/MIP-3A/MIP-3[a]/MIP3A/ST38/Scya2(	PPM03142B
B01	Mm.12895	NM_009137	Ccl22	Chemokine (C-C motif) ligand 22	ABCD-1/DCBCK/MDC/Scya22	PPM02950B
B02	Mm.31505	NM_019577	Ccl24	Chemokine (C-C motif) ligand 24	CKb-6/MPIF-2/Scya24	PPM03159F
B03	Mm.1282	NM_011337	Ccl3	Chemokine (C-C motif) ligand 3	AI323804/G0S19-1/LD78alpha/MIP-1alpha/MIP1-	PPM02949F
B04	Mm.244263	NM_013652	Ccl4	Chemokine (C-C motif) ligand 4	AT744.1/Act-2/MIP-1B/Mip1b/Scya4	PPM02948F
B05	Mm.284248	NM_013653	Ccl5	Chemokine (C-C motif) ligand 5	MuRantes/RANTES/SISd/Scya5/TCP228	PPM02960F
B06	Mm.341574	NM_013654	Ccl7	Chemokine (C-C motif) ligand 7	MCP-3/Scya7/fic/marc/mcp3	PPM02955B
B07	Mm.4861	NM_011616	Cd40lg	CD40 ligand	CD154/CD40-L/Cd40l/HIGM1/IGM/IMD3/Ly-62/Ly	PPM03226C
B08	Mm.42228	NM_011617	Cd70	CD70 antigen	CD27LG/Cd27l/Tnfsf7	PPM03552E
B09	Mm.290924	NM_170786	Cntf	Ciliary neurotrophic factor	AI429687	PPM68695B
B10	Mm.795	NM_007778	Csf1	Colony stimulating factor 1 (macrophage)	C87615/Csfm/MCSF/op	PPM03116C
B11	Mm.4922	NM_009969	Csf2	Colony stimulating factor 2 (granulocyte-ma	Csfgm/GMCSF/Gm-CSf/MGI-IGM	PPM02990F
B12	Mm.1238	NM_009971	Csf3	Colony stimulating factor 3 (granulocyte)	Csfg/G-CSF/MGI-IG	PPM02989B
C01	Mm.389954	NM_007795	Ctf1	Cardiotrophin 1	CT-1	PPM05017D
C02	Mm.103711	NM_009142	Cx3cl1	Chemokine (C-X3-C motif) ligand 1	AB030188/ABCD-3/AI848747/CX3C/Cxc3/D8Bw	PPM02959F
C03	Mm.21013	NM_008176	Cxcl1	Chemokine (C-X-C motif) ligand 1	Fsp/Gro1/KC/Mgsa/N51/Scyb1/gro	PPM03058C
C04	Mm.877	NM_021274	Cxcl10	Chemokine (C-X-C motif) ligand 10	C7/CRG-2/INP10/IP-10/IP10/Ifi10/Scyb10/gIP-10/	PPM02978E
C05	Mm.131723	NM_019494	Cxcl11	Chemokine (C-X-C motif) ligand 11	Cxc11/H174/I-tac/Ip9/Itac/Scyb11/Scyb9b/b-R1/b	PPM03192C
C06	Mm.303231	NM_021704	Cxcl12	Chemokine (C-X-C motif) ligand 12	Pbsf/Scyb12/Sdf1/Tlsf/Tpar1	PPM02965E
C07	Mm.10116	NM_018866	Cxcl13	Chemokine (C-X-C motif) ligand 13	4631412M08Rik/ANGIE2/Angie/BCA-1/BLC/BLR	PPM02947G
C08	Mm.425692	NM_023158	Cxcl16	Chemokine (C-X-C motif) ligand 16	0910001K24Rik/AV290116/BB024863/CXCL16v	PPM03775A
C09	Mm.244289	NM_203320	Cxcl3	Chemokine (C-X-C motif) ligand 3	Dcip1/Gm1960	PPM34590C
C10	Mm.4660	NM_009141	Cxcl5	Chemokine (C-X-C motif) ligand 5	AMCF-II/ENA-78/GCP-2/LIX/Scyb5/Scyb6	PPM02966F
C11	Mm.766	NM_008599	Cxcl9	Chemokine (C-X-C motif) ligand 9	BB139920/CMK/Mig/MuMIG/Scyb9/crg-10	PPM02973B
C12	Mm.3355	NM_010177	Fasl	Fas ligand (TNF superfamily, member 6)	APT1LG1/CD178/CD95-L/CD95L/Fas-L/Faslg/Tn	PPM02926E
D01	Mm.589	NM_008155	Gpi1	Glucose phosphate isomerase 1	Amf/Gpi/Gpi-1/Gpi-1r/Gpi-1s/Gpi-1t/Gpi1-r/Gpi1-s/	PPM03345C
D02	Mm.2168	NM_010406	Hc	Hemolytic complement	C5/C5a/He	PPM24764C
D03	Mm.14091	NM_010503	Ifna2	Interferon alpha 2	Ifa2	PPM03543A
D04	Mm.240327	NM_008337	Ifng	Interferon gamma	IFN-g/Ifg	PPM03121A
D05	Mm.874	NM_010548	Il10	Interleukin 10	CSIF/Il-10	PPM03017C
D06	Mm.35814	NM_008350	Il11	Interleukin 11	IL-11	PPM03018E
D07	Mm.103783	NM_008351	Il12a	Interleukin 12A	IL-12p35/Il-12a/Ll12a/p35	PPM03019A
D08	Mm.239707	NM_008352	Il12b	Interleukin 12B	Il-12b/Il-12p40/Il12p40/p40	PPM03020E
D09	Mm.1284	NM_008355	Il13	Interleukin 13	Il-13	PPM03021B
D10	Mm.490053	NM_008357	Il15	Interleukin 15	AI503618/IL-15	PPM03022C
D11	Mm.10137	NM_010551	Il16	Interleukin 16	mKIAA4048	PPM03111E
D12	Mm.5419	NM_010552	Il17a	Interleukin 17A	Ctla-8/Ctla8/IL-17/IL-17A/Il17	PPM03023A
E01	Mm.222807	NM_145856	Il17f	Interleukin 17F	C87042/IL-17F	PPM05398E
E02	Mm.1410	NM_008360	Il18	Interleukin 18	Igif/Il-18	PPM03112B
E03	Mm.15534	NM_010554	Il1a	Interleukin 1 alpha	Il-1a	PPM03010F
E04	Mm.222830	NM_008361	Il1b	Interleukin 1 beta	IL-1beta/Il-1b	PPM03109F
E05	Mm.882	NM_031167	Il1rn	Interleukin 1 receptor antagonist	F630041P17Rik/IL-1ra	PPM03547B
E06	Mm.14190	NM_008366	Il2	Interleukin 2	Il-2	PPM02937C
E07	Mm.157689	NM_021782	Il21	Interleukin 21	-	PPM03761F
E08	Mm.103585	NM_016971	Il22	Interleukin 22	IL-22/IL-22a/ILTIFa/Iltif	PPM05481A
E09	Mm.125482	NM_031252	Il23a	Interleukin 23, alpha subunit p19	IL-23/p19	PPM03763F
E10	Mm.196691	NM_053095	Il24	Interleukin 24	FISP/Mda-7/Mda7/St16	PPM04205C
E11	Mm.222632	NM_145636	Il27	Interleukin 27	IL-27/IL-27p28/Il30/p28	PPM33809A
E12	Mm.983	NM_010556	Il3	Interleukin 3	BPA/Csfmu/HCGF/Il-3/MCGF/PSF	PPM03012F
F01	Mm.276360	NM_021283	Il4	Interleukin 4	BSF-1/Il-4	PPM03013F
F02	Mm.4461	NM_010558	Il5	Interleukin 5	Il-5	PPM03014F
F03	Mm.1019	NM_031168	Il6	Interleukin 6	Il-6	PPM03015A
F04	Mm.3825	NM_008371	Il7	Interleukin 7	A630026I06Rik/Il-7/hlb368	PPM03016C

F05	Mm.3006	NM_008373	Il9	Interleukin 9	Il-9/P40	PPM03110A
F06	Mm.4964	NM_008501	Lif	Leukemia inhibitory factor	-	PPM02988F
F07	Mm.87787	NM_010735	Lta	Lymphotoxin A	LT/LT-[a]/LT-alpha/LT[a]/LTalpha/Ltx/TNF-beta/TN	PPM03114A
F08	Mm.1715	NM_008518	Ltb	Lymphotoxin B	AI662801/LTbeta/Tnfc/Tnfsf3/p33	PPM03119A
F09	Mm.2326	NM_010798	Mif	Macrophage migration inhibitory factor	GIF/Glif	PPM02985H
F10	Mm.3514	NM_010834	Mstn	Myostatin	Cmpt/Gdf8	PPM04441F
F11	Mm.57195	NM_013611	Nodal	Nodal	Tg.413d	PPM04458A
F12	Mm.131422	NM_0010133	Osm	Oncostatin M	OncoM	PPM05385B
G01	Mm.332490	NM_019932	Pf4	Platelet factor 4	Cxcl4/Scyb4	PPM02986C
G02	Mm.293614	NM_023785	Ppbp	Pro-platelet basic protein	2400003M24Rik/AI854500/CTAP3/CTAPIII/Cxcl7	PPM03773A
G03	Mm.288474	NM_009263	Spp1	Secreted phosphoprotein 1	2AR/Apl-1/BNSP/BSPI/Bsp/ETA-1/Eta/OP/Opn/Op	PPM03648C
G04	Mm.18213	NM_009367	Tgfb2	Transforming growth factor, beta 2	BB105277/Tgf-beta2/Tgfb-2	PPM02992A
G05	Mm.3943	NM_009379	Thpo	Thrombopoietin	Mgdf/Ml/Mpllg/Tpo/Tpo1/Tpo2/Tpo3/Tpo4	PPM02984B
G06	Mm.1293	NM_013693	Tnf	Tumor necrosis factor	DIF/TNF-a/TNF-alpha/TNFSF2/TNFalpha/Tnfa/Tn	PPM03113G
G07	Mm.15383	NM_008764	Tnfrsf11b	Tumor necrosis factor receptor superfamily,	OCIF/Opg/TR1	PPM03404F
G08	Mm.1062	NM_009425	Tnfsf10	Tumor necrosis factor (ligand) superfamily, r	A330042I21Rik/AI448571/APO-2L/Ly81/TL2/Trail	PPM02925B
G09	Mm.249221	NM_011613	Tnfsf11	Tumor necrosis factor (ligand) superfamily, r	Ly109l/ODF/OPG/OPGL/RANKL/Trance	PPM03047F
G10	Mm.28835	NM_033622	Tnfsf13b	Tumor necrosis factor (ligand) superfamily, r	BAFF/BLyS/D8Ertd387e/TALL-1/TALL1/THANK/T	PPM03751B
G11	Mm.282184	NM_009505	Vegfa	Vascular endothelial growth factor A	Vegf/Vpf	PPM03041F
G12	Mm.190	NM_008510	Xcl1	Chemokine (C motif) ligand 1	AI661682/ATAC/LTN/Lptn/SCM-1/SCM-1a/Scyc1	PPM02956C
H01	Mm.391967	NM_007393	Actb	Actin, beta	Actx/E430023M04Rik/beta-actin	PPM02945B
H02	Mm.163	NM_009735	B2m	Beta-2 microglobulin	Ly-m11/beta2-m/beta2m	PPM03562A
H03	Mm.304088	NM_008084	Gapdh	Glyceraldehyde-3-phosphate dehydrogenas	Gapd	PPM02946E
H04	Mm.3317	NM_010368	Gusb	Glucuronidase, beta	AI747421/Gur/Gus/Gus-r/Gus-s/Gus-t/Gus-u/Gut/a	PPM05490C
H05	Mm.2180	NM_008302	Hsp90ab1	Heat shock protein 90 alpha (cytosolic), clas	90kDa/AL022974/C81438/Hsp84/Hsp84-1/Hsp90	PPM04803F
H06	N/A	SA_00106	MGDC	Mouse Genomic DNA Contamination	MIGX1B	
H07	N/A	SA_00104	RTC	Reverse Transcription Control	RTC	PPX63340A
H08	N/A	SA_00104	RTC	Reverse Transcription Control	RTC	PPX63340A
H09	N/A	SA_00104	RTC	Reverse Transcription Control	RTC	PPX63340A
H10	N/A	SA_00103	PPC	Positive PCR Control	PPC	
H11	N/A	SA_00103	PPC	Positive PCR Control	PPC	
H12	N/A	SA_00103	PPC	Positive PCR Control	PPC	

Table S2. PCR primers and probes for qRT-PCR.

	Gene Symbol	Unigene	Primers	Nucleotide sequences	Probe number
1	*Cxcl9*	NM_008599.4	Forward	cttttcctcttgggcatcat	1
			Reverse	gcatcgtgcattccttatca	
2	*Cxcl10*	NM_021274	Forward	gctgccgtcattttctgc	3
			Reverse	tctcactggcccgtcatc	
3	*Ccl5*	NM_013653.3	Forward	tgcagaggactctgagacagc	10
			Reverse	gagtggtgtccgagccata	
4	*Ifng*	NM_008337	Forward	atctggaggaactggcaaaa	21
			Reverse	ttcaagacttcaaagagtctgaggta	
5	*Cxcr3*	NM_009910.2	Forward	aggcagcacgagacctga	66
			Reverse	ggcatctagcacttgacgttc	
6	*Ccl17*	NM_011332.3	Forward	tgcttctggggacttttctg	27
			Reverse	gaatggccccttgaagtaa	
7	*Ccl22*	NM_009137.2	Forward	tcttgctgtggcaattcaga	84
			Reverse	gagggtgacggatgtagtcc	
8	*Il4*	NM_021283	Forward	catcggcattttgaacgag	2
			Reverse	cgagctcactctctgtggtg	
9	*Il5*	NM_010558.1	Forward	acattgaccgccaaaaagag	91
			Reverse	atccaggaactgcctcgtc	
10	*Il10*	NM_010548	Forward	cagagccacatgctcctaga	41
			Reverse	tgtccagctggtcctttgtt	
11	*Ccr4*	NM_009916.2	Forward	ctcaggatcactttcagaagagc	18
			Reverse	ggcattcatctttggaatcg	
12	*Foxp3*	NM_001199347.1	Forward	aagtggcagagaggtattgagg	92
			Reverse	cttctctaagctttcttctgtctgg	
13	*Gata3*	NM_008091.3	Forward	ttatcaagcccaagcgaag	108
			Reverse	tggtggtggtctgacagttc	
14	*Rn18s*	NR_003278	Forward	gcaattattccccatgaacg	48
			Reverse	gggacttaatcaacgcaagc	
